# The Crucial Involvement of Retinoid X Receptors in DDE Neurotoxicity

**DOI:** 10.1007/s12640-015-9572-6

**Published:** 2015-11-13

**Authors:** A. Wnuk, J. Rzemieniec, E. Litwa, W. Lasoń, W. Krzeptowski, A. K. Wójtowicz, M. Kajta

**Affiliations:** Department of Experimental Neuroendocrinology, Institute of Pharmacology, Polish Academy of Sciences, 12 Smetna Street, 31-343 Kraków, Poland; Department of Genetics and Evolution, Institute of Zoology, Jagiellonian University, 9 Gronostajowa Street, 30-387 Kraków, Poland; Department of Animal Biotechnology, Faculty of Animal Sciences, University of Agriculture, Redzina 1B, 30-248 Kraków, Poland

**Keywords:** Neurotoxicity, Retinoid X receptor, RXR, DDE, DDT, Primary neuronal cell cultures

## Abstract

Dichlorodiphenyldichloroethylene (DDE) is a primary environmental and metabolic degradation product of the pesticide dichlorodiphenyltrichloroethane (DDT). It is one of the most toxic compounds belonging to organochlorines. DDE has never been commercially produced; however, the parent pesticide DDT is still used in some developing countries for disease-vector control of malaria. DDT and DDE remain in the environment because these chemicals are resistant to degradation and bioaccumulate in the food chain. Little is known, however, about DDE toxicity during the early stages of neural development. The results of the present study demonstrate that DDE induced a caspase-3-dependent apoptosis and caused the global DNA hypomethylation in mouse embryonic neuronal cells. This study also provided evidence for DDE-isomer-non-specific alterations of retinoid X receptor α (RXRα)- and retinoid X receptor β (RXRβ)-mediated intracellular signaling, including changes in the levels of the receptor mRNAs and changes in the protein levels of the receptors. DDE-induced stimulation of RXRα and RXRβ was verified using selective antagonist and specific siRNAs. Co-localization of RXRα and RXRβ was demonstrated using confocal microscopy. The apoptotic action of DDE was supported at the cellular level through Hoechst 33342 and calcein AM staining experiments. In conclusion, the results of the present study demonstrated that the stimulation of RXRα- and RXRβ-mediated intracellular signaling plays an important role in the propagation of DDE-induced apoptosis during early stages of neural development.

## Introduction

Dichlorodiphenyldichloroethylene (DDE) is a toxic organochlorine compound and the primary environmental and metabolic degradation product of the pesticide DDT. DDE has never been produced commercially; however, the parent pesticide DDT was commonly used for insect control until the use of this compound was canceled for agricultural use worldwide in the 1970’s. Although DDT is no longer produced in most Western countries, the insecticide is still used in some developing countries for disease-vector control of malaria. DDT and DDE remain in the environment because these compounds are resistant to degradation and bioaccumulate in the food chain. Commercial DDT is a mixture of closely related isomers—*p,p*′-DDT (~80 %) and *o,p*′-DDT (~20 %) that metabolize to *p,p*′- and *o,p*′-DDE, respectively, which are more stable than DDT. DDE is a highly fat-soluble compound that accumulates in the fatty tissues of insects, wildlife, and humans, particularly in adipose and brain tissues (Escuder-Gilabert et al. 2009). Studies have shown that in patients with Alzheimer’s disease, serum levels of DDE are significantly higher (almost four times) than in controls (Richardson et al. 2014). Furthermore, Chhillar et al. (2013) observed an increase in the serum levels of *p,p′*-DDE in Parkinson’s disease subjects.

Increasing evidence suggests that the presence of DDE in the development induces brain damage at doses much lower than those affecting adult brain function, leading to disruption of fetal neurodevelopmental processes. The effects of in utero exposure to DDE investigated among the Mexican–American farmworker population revealed a negative association with mental and psychomotor development at 12 and 24 months (Eskenazi et al. 2006). A recent epidemiological study showed that DDE prenatal exposure has a long-term effect and is negatively associated with child neural development at 3.5–5 years of age (Torres-Sánchez et al. 2013). The impact of *p,p′*-DDT or *p,p′*-DDE exposure on sensory development is unknown; however, exposure to *p,p′*-DDE both pre- and during the first postnatal years might undermine visual processing in pre-adolescent children (Cartier et al. 2014). Exposure to DDE has been associated with impaired cognitive skills in both 4-year-old children and elderly individuals (Ribas-Fitó et al. 2006; Kim et al. 2015). A Finnish study showed that prenatal exposure to high levels of DDE was associated with increased risk for autism in offspring (Cheslack-Postava et al. 2013). *In utero* DDE exposure has also been associated with ADHD-like behaviors in children 7–11 years of age (Sagiv et al. 2010).

The retinoid X receptor (RXR) is an intriguing and essential member of a nuclear receptor superfamily encoded by 3 distinct genes: RXRα, RXRβ, and RXRγ (Evans et al. 2014). RXRs were initially identified as heterodimeric partners of retinoic acid receptor (RAR), thyroid hormone receptor (T3R), and vitamin D receptor (VDR). Currently, RXRs have been described as heterodimers with approximately one-third of the 48 human nuclear receptor superfamily members, including Nur77, peroxisome proliferator-activated receptors (PPARs), liver X receptor (LXR), and farnesoid X receptor (FXR) (Rőszer et al. 2013). Most of nuclear receptors require RXR as an obligatory partner for DNA binding and transcriptional regulation. In addition, RXRα forms homodimers and homotetramers, suggesting the self-regulation of specific RXRα signaling pathways (Zhang et al. 2011). RXRs have several endogenous ligands, such as 9-*cis*-retinoic acid (RA), docosahexaenoic acid (DHA), oleic acid, and phytanic acid; however, none of compounds have been demonstrated as *bona fide* interacting partners. Organotin compounds (e.g., tributyltin (TBT), triphenyltin (TPT)) act as RXR agonists with strong effects on these receptors at levels comparable to those of 9-*cis* retinoic acid (Kanayama et al. 2005). The diversity of RXRs suggests an important role for these molecules as regulators of a wide range of cellular pathways.

According to current data, the role of RXRs in neuronal survival is complicated. Many papers indicate that RXR activation improves neuronal survival in animal models of Parkinson’s disease and amyotrophic lateral sclerosis (McFarland et al. 2013; Riancho et al. 2015, Esteves et al. 2015). These neuroprotective actions are frequently suggested to be mediated by Nurr1-RXR heterodimers (Wallen-Mackenzie et al. 2003). There are, however, other RXR-containing heterodimers, which are postulated to exhibit pro-apoptotic capacity. Nur77-RXR heterodimers have been linked to the induction of apoptosis in LNCaP prostate cancer cells and in H460 lung cancer cells (Cao et al. 2004). Therefore, depending on heterodimerization partner, RXR agonists and antagonists may cause pro- or anti-apoptotic effects. In addition, Qin et al. (2008) delineated the regions of RXRα that are required for growth inhibition and apoptosis, including RXR-dependent caspase activation. Bexarotene is an RXR agonist and anti-tumor agent which has been approved for the treatment of refractory or persistent cutaneous T cell lymphoma via apoptosis induction.

Studies have shown the importance of 9-*cis* retinoid acid–RXR signaling in regulating dopaminergic and cholinergic innervation in health and disease, e.g., Parkinson’s disease, mental disorders, and extrapyramidal motor tract dysfunctions (Huang et al. 2011). Individuals suffering from dementia exhibit higher levels of RXRα gene and protein expression in the inferior temporal gyrus (Akram et al. 2010). Moreover, treatment with RXR agonists (bexarotene and fluorobexarotene) increases amyloid-beta clearance in vivo and in vitro (Bachmeier et al. 2013). A previous study demonstrated that RXR is expressed during motor neuron degeneration in an amyotrophic lateral sclerosis (ALS) rat model (Jokic et al. 2007). Retinoid signaling has also been implicated in disorders of the nervous system, such as schizophrenia and depression (Goodman 1998; Wysowski et al. 2001). Dysfunctional retinoid signaling induces cognitive impairments (McCaffery et al. 2006). RXRα or RXRβ deficiencies in mice are embryolethal; however, RXRγ-knockdown mice survive and appear normal. The upregulation of RXRβ is a characteristic response of astroglial activation under circumstances of neural damage. Long-term potentiation (LTP) and long-term depression (LTD) are dependent on retinoid signaling, and vitamin A deficiency leads to impaired learning and memory (Chiang et al. 1998; Misner et al. 2001; Cocco et al. 2002; Etchamendy et al. 2003). Recent analysis of RXRγ knockout mice has shown an impact on oligodendrocyte differentiation, spatial learning, and memory function (Huang et al. 2011; Nomoto et al. 2012).

In a recent study, we demonstrated that RXR mediates the apoptotic effects of 4-*para*-nonylphenol (NP) in mouse embryonic neuronal cells (Litwa et al. 2014). The aim of present study was to investigate the role of RXR in DDE-induced apoptotic and neurotoxic effects. Herein, we evaluated the effects of *p,p′*-DDE and *o,p′*-DDE on apoptotic and neurotoxic parameters, such as caspase-3 activation and lactate dehydrogenase (LDH) release. To assess whether the actions of DDE are tissue-dependent, we examined hippocampal, neocortical, and cerebellar tissues. The cellular analyses included Hoechst 33342 and calcein AM staining experiments to visualize apoptotic DNA-fragmentation and assess cell survival, respectively. To verify the involvement of particular RXRs in DDE-mediated neurotoxicity, potent RXR antagonist and specific siRNAs were employed. In addition, the mRNA expression levels of *Rxrα, Rxrβ*, and *Rxrγ* were measured using qPCR. The levels of the protein receptors were detected using ELISA and Western blotting. Quantification of global DNA methylation was based on detection of methylated cytosines. The neuronal distribution of RXRα and RXRβ was demonstrated with immunofluorescent labeling and confocal microscopy.

## Materials and Methods

### Materials

B27 and Neurobasal media were obtained from Gibco (Grand Island, NY, USA). l-glutamine, fetal bovine serum (FBS), *N*-acetyl-Asp-Glu-Val-Asp *p*-nitro-anilide (Ac-DEVD-*p*NA), dimethyl sulfoxide (DMSO), HEPES, CHAPS, mouse monoclonal anti-MAP2 antibody, ammonium persulfate, TEMED, TRIZMA base, Tween 20, DL-dithiothreitol, Nonidet NP-40, sodium deoxycholate, protease inhibitor (EDTA-free), Imprint^®^ Methylated DNA Quantification, bromophenol blue, and poly-ornithine were obtained from Sigma-Aldrich (St. Louis, MO, USA). Bradford reagent, SDS, 30 % acrylamide, 0.5 M Tris–HCl buffer, 1.5 M Tris–HCl gel buffer, and Laemmli Sample Buffer were from BioRad Laboratories (Munchen, Germany), and HX 531 was from Tocris Bioscience (Minneapolis, MN, USA). 2-mercaptoethanol was from Carl Roth GmbH + Co. KG, (Karlsruhe, Germany). Immobilon-P membranes were purchased from Millipore (Bedford, MA, USA). Calcein AM and Hoechst 33342 were purchased from Molecular Probes (Eugene, OR, USA). Cy3-conjugated anti-rabbit IgG and Cy5-conjugated anti-mouse were obtained from Jackson ImmunoResearch, Inc. (West Grove, PA, USA). The Cytotoxicity Detection Kit and BM chemiluminescence Western blotting substrate (POD) were purchased from Roche Diagnostics GmbH (Mannheim, Germany); ELISA assay kits for RXRα and RXRβ were purchased from USCN Life Science Inc. (Wuhan, China). The culture dishes were obtained from TPP Techno Plastic Products AG (Trasadingen, Switzerland). Rabbit polyclonal anti-RXRα antibody (sc-774), mouse monoclonal anti-RXRβ antibody (sc-56869), mouse monoclonal anti β-Actin antibody (sc-47778), RXRα siRNA (sc-36448), and RXRβ siRNA (sc-36446) were purchased from Santa Cruz Biotechnology, Inc. (Santa Cruz, CA, USA). AllStars Negative Control siRNA AF 488 and the RNeasy Mini Kit were obtained from Qiagen (Valencia, CA, USA). INTERFERin was obtained from PolyPlus Transfection (Illkirch, France), and the High Capacity cDNA-Reverse Transcription Kit, the TaqMan Gene Expression Master Mix, and TaqMan probes corresponding to specific genes encoding *Hprt*, *Rxrα,Rxrβ,* and *Rxrγ* were obtained from Life Technologies Applied Biosystems (Foster City, CA, USA).

### Primary Neocortical, Hippocampal, and Cerebellar Neuronal Cell Cultures

Neocortical, hippocampal, and cerebellar tissues for primary cultures were prepared from Swiss mouse embryos (Charles River, Germany) at 15–17 days of gestation and cultured as previously described (Kajta et al. 2007). In the case of cerebellar cultures, the tissue originated from 7-day-old mouse pups to obtain cultures enriched in granule cells. All procedures were performed in accordance with the National Institutes of Health Guidelines for the Care and Use of Laboratory Animals and approved through the Bioethics Commission in compliance with Polish Law (21 August 1997). Animal care followed official governmental guidelines, and all efforts were made to minimize suffering and the number of animals used. The cells were suspended in estrogen-free neurobasal medium with B27 supplement on poly-ornithine (0.01 mg per ml)-coated multi-well plates at a density of 2.0 × 10^5^ cells per cm^2^. Cerebellar cultures were supplemented with 25 mM KCl. The cultures were maintained at 37 °C in a humidified atmosphere containing 5 % CO_2_ for 7 days in vitro (DIV) prior to experimentation. The amount of astrocytes, as determined by the content of intermediate filament protein GFAP (glial fibrillary acidic protein), did not exceed 10 % for all cultures (Kajta et al. 2009).

### Treatment

Primary neuronal cell cultures were exposed to *p,p*′-DDE (0.1–100 μM) and *o,p*′-DDE (0.1–100 μM) for 6 or 24 h. To assess whether the effects of DDE were tissue-dependent, we examined these effects in hippocampal, neocortical, and cerebellar cultures. The role of the RXRs in the actions of DDE was assessed via pretreatment with the potent RXR antagonist HX 531 (0.1 μM). To avoid non-specific effects, selective receptor ligand was used at a concentration that did not affect the control levels of caspase-3 activity and LDH release. All the compounds were originally dissolved in DMSO and further diluted in culture medium to maintain the DMSO concentration below 0.1 %. The control cultures were treated with DMSO at concentrations equal to those used in the experimental groups.

### Identification of Apoptotic Cells

Apoptotic cells were detected via Hoechst 33342 staining at 24 h after the initial treatment, as previously described (Kajta et al. 2007). Hippocampal cells cultured on glass cover slips were washed with 10 mM phosphate-buffered saline (PBS) and exposed to Hoechst 33342 (0.6 mg/ml) staining at room temperature (RT) for 5 min. The cells containing bright blue fragmented nuclei, indicating condensed chromatin, were identified as apoptotic cells. Qualitative analysis was performed using a fluorescence microscope (NIKON Eclipse 80i, NIKON Instruments Inc., Melville, New York, USA) equipped with a camera with BCAM Viewer copyright Basler AG software.

### Staining with Calcein AM

Measurement of intracellular esterase activity in hippocampal cultures was based on calcein AM staining at 24 h after initial treatment (Kajta et al. 2007). To avoid the esterase activity present in the growth media, the cells were washed with PBS and incubated in 2 μM calcein AM in PBS at RT for 10 min. The cells displaying a bright green cytoplasm were identified as living cells. Fluorescence intensity was monitored at Ex/Em 494/520 nm using a fluorescence microscope (NIKON Eclipse 80i, NIKON Instruments Inc., Melville, New York, USA) equipped with a camera with BCAM Viewer copyright Basler AG software.

### Assessment of Caspase-3 Activity

Caspase-3 activity was determined according to Nicholson et al. (1995), using samples treated for 6 or 24 h with *p,p*′-DDE or *o,p*′-DDE alone or in combination with the test compounds. The assessment of caspase-3 activity was performed as previously described (Kajta et al. 2009). Cell lysates from hippocampal, neocortical, and cerebellar cultures were incubated at 37 °C using a colorimetric substrate preferentially cleaved by caspase-3, called Ac-DEVD-*p*NA (*N*-acetyl-asp-glu-val-asp-*p*-nitro-anilide). The levels of *p*-nitroanilide were continuously monitored for 60 min using a Multimode Microplate Reader Infinite M200PRO (Tecan, Mannedorf, Switzerland). The data were analyzed using Magellan software, normalized to the absorbency of vehicle-treated cells, and expressed as a percentage of control ± SEM from three to four independent experiments. The absorbance of blanks, acting as no-enzyme controls, was subtracted from each value.

### Measurement of Lactate Dehydrogenase Activity

To quantify cell death, lactate dehydrogenase (LDH) release from damaged cells into the cell culture media was measured 6 or 24 h after treatment with *p,p*′-DDE or *o,p*′-DDE. LDH release was measured as previously described (Kajta et al. 2005). Cell-free supernatants from hippocampal, neocortical, and cerebellar cultures were collected from each well and incubated at room temperature for 30–60 min with the appropriate reagent mixture according to the manufacturer’s instructions (Cytotoxicity Detection Kit) depending on reaction progress. The intensity of the red color formed in the assay, measured at a wavelength of 490 nm (Infinite M200pro microplate reader, Tecan Mannedorf, Switzerland), was proportional to both LDH activity and the number of damaged cells. The data were analyzed using i-control™ software, normalized to the color intensity from vehicle-treated cells (100 %), and expressed as a percentage of the control value from three to four independent experiments. The absorbance of blanks, acting as no-enzyme controls, was subtracted from each value.

### Silencing of RXRα and RXRβ

Specific siRNAs were used to inhibit RXRα and RXRβ expressions in hippocampal cells. Each siRNA was applied separately for 6 h at 50 nM in antibiotic-free medium containing the siRNA transfection reagent INTERFERin™. After transfection, the culture media were changed, and the cells were incubated for 24 h before starting the experiment. Positive and negative siRNAs containing a scrambled sequence that did not lead to the specific degradation of any known cellular mRNA were used as controls. The effectiveness of mRNA silencing was verified through the measurement of specific mRNAs using qPCR.

### Measurement of Global DNA Methylation

Global DNA methylation shifts at 24 h after treatment with *p,p*′-DDE or *o,p*′-DDE were measured in hippocampal cells using a specific ELISA-based format kit (Imprint^®^ Methylated DNA Quantification—Sigma-Aldrich; St. Louis, MO, USA). This kit contains all reagents required to detect relative levels of methylated DNA. Quantification of global DNA methylation was obtained from calculating the amount of methylated cytosines in each sample relative to global cytidine in a positive control i.e. the sample that had been previously methylated. The methylated DNA was detected using the capture and detection antibodies and quantified colorimetrically using an Infinite M200pro microplate reader (Tecan, Austria). The amount of methylated DNA present in the sample is proportional to the absorbance measured.

### qPCR Analysis of mRNAs Specific to Genes Encoding the Receptors *Rxrα, Rxrβ*, and *Rxrγ*

Total RNA was extracted from hippocampal cells cultured for 7 DIV (approx. 1.5 × 10^6^ cells per sample) using the RNeasy Mini Kit (Qiagen, Valencia, CA) according to the manufacturer’s instructions. The quantity of RNA was spectrophotometrically determined at 260 and 260/280 nm (ND/1000 UV/Vis; Thermo Fisher NanoDrop, USA). Two-step real-time RT-PCR was performed. Both the reverse transcription (RT) reaction and quantitative polymerase chain reaction (qPCR) were run in the CFX96 Real-Time System (BioRad, USA). The products of the RT reaction were amplified using the TaqMan Gene Expression Master Mix containing TaqMan primer probes specific to the genes encoding *Hprt*, *Rxrα, Rxrβ, and Rxrγ.* Amplification was performed in a total volume of 20 µl of the mixture containing 10 µl of the TaqMan Gene Expression Master Mix and 1.0 µl of the RT product as the PCR template. A standard qPCR procedure was performed: 2 min at 50 °C and 10 min at 95 °C followed by 40 cycles of 15 s at 95 °C and 1 min at 60 °C. The threshold value (Ct) for each sample was set during the exponential phase, and the delta delta Ct method was used for data analysis. *Hprt* (hypoxanthine phosphoribosyltransferase coding gene) was used as a reference gene.

### Western Blot Analysis

The cells exposed for 24 h to *p,p*′-DDE or *o,p*′-DDE were lysed in ice-cold lysis buffer containing 50 mM Tris–HCl, pH 7.5, 100 mM NaCl, 0.5 % sodium deoxycholate, 0.5 % octylphenoxypolyethoxyethanol (IGEPAL CA-630), EDTA-free protease inhibitors, and 0.5 % SDS. The lysates were sonicated and centrifuged at 15,000×*g* for 20 min at 4 °C. The protein concentrations in the supernatants were determined using Bradford reagent (BioRad Protein Assay) with bovine serum albumin (BSA) as the standard. Samples containing 40 μg of total protein were reconstituted in the appropriate amount of sample buffer comprising 125 mM Tris, pH 6.8, 4 % SDS, 25 % glycerol, 4 mM EDTA, 20 mM DTT, and 0.01 % bromophenol blue, denatured and separated on 7.5 % SDS-polyacrylamide gel using a BioRad Mini-Protean II electrophoresis cell, as previously described (Wójtowicz et al. 2007). After electrophoretic separation, the proteins were electrotransferred to PVDF membranes (Millipore, Bedford, MA, USA) using the BioRad Mini Trans-Blot apparatus. Following the transfer, the membranes were washed, and non-specific binding sites were blocked with 5 % dried milk and 0.2 % Tween-20 in 0.02 M TBS (Tris-buffered saline) for 2 h with shaking. The membranes were incubated overnight (at 4 °C) with one of the following primary antibodies (Santa Cruz Biotechnology): anti-RXRα rabbit polyclonal antibody (diluted 1:100), anti-RXRβ mouse polyclonal antibody (diluted 1:100), or anti-β-actin mouse monoclonal antibody (diluted 1:3000) diluted in TBS/Tween. To control the amount of denatured protein loaded onto the gel, the membranes were stripped and reprobed with an anti-β-actin antibody (Santa Cruz Biotechnology). The signals were developed by chemiluminescence (ECL) using BM Chemiluminescence Blotting Substrate (Roche Diagnostics GmBH) and visualized with Luminescent Image Analyzer Fuji-Las 4000 (Fuji, Japan). Immunoreactive bands were quantified using an image analyzer MultiGauge V3.0, as previously described (Rzemieniec et al. 2015).

### Enzyme-Linked Immunosorbent Assays for RXRα and RXRβ

The levels of RXRα and RXRβ were determined in hippocampal cells 24 h after treatment with *p,p*′-DDE and *o,p*′-DDE. Specific detection of these proteins was obtained using enzyme-linked immunosorbent assays (ELISAs) and the quantitative sandwich enzyme immunoassay technique. A 96-well plate was pre-coated with monoclonal antibodies specific to RXRα and RXRβ. The standards and non-denatured cell extracts were added to the wells with biotin-conjugated polyclonal antibodies specific for RXRα or RXRβ. Therefore, all native RXRα or RXRβ proteins were captured using the immobilized antibodies. The plates were washed to remove any unbound substances, and horseradish peroxidase-conjugated avidin was added to interact with the biotin bound to RXRα and RXRβ. After washing, the substrate solution was added to the wells. The enzyme reaction yielded a blue product. The absorbance was measured at 450 nm and was proportional to the amount of RXRα or RXRβ. The protein concentration was determined in each sample with Bradford reagent (BioRad Protein Assay).

### Immunofluorescent Labeling of RXRα and RXRβ and Confocal Microscopy

For immunofluorescence detection of RXRα and RXRβ, hippocampal cells were grown on glass cover slips and subjected to immunofluorescence double-labeling, as previously described (Kajta et al. 2013). After a 1-h incubation in a blocking buffer (5 % normal donkey serum and 0.3 % Triton X-100 in 0.01 M PBS), the cells were treated for 24 h (at 4 °C) using three primary antibodies: anti-RXRα rabbit polyclonal (1:50), anti-RXRβ mouse monoclonal (1:50), and anti-MAP2 mouse monoclonal (1:100), followed by a 24-h incubation in a mixture of secondary antibodies, including Cy3-conjugated anti-rabbit IgG (1:300) and Cy5-conjugated anti-mouse IgG (1:300). The samples were subsequently washed, mounted, cover-slipped, and analyzed using a confocal laser scanning microscope LSM510 META, Axiovert 200 M (Carl Zeiss MicroImaging GmbH, Jena, Germany) under a Plan-Neofluar 40×/1.3 Oil DIC objective. A He/Ne laser and an argon laser, with two laser lines emitting at 514 and 633 nm, were used to excite the Cy3- and Cy5-conjugated antibodies, respectively. The fluorescence signal was enhanced after summing four scans per line. A pinhole value of 1 airy unit was used to obtain flat images.

### Data Analysis

Statistical tests were performed on raw data expressed as the mean arbitrary absorbance or fluorescence units per well containing 50,000 cells (measurements of caspase-3, LDH), the fluorescence units per 1.5 million cells (PCR), the absorbance units per 150 ng DNA (DNA methylation) and the mean optical density per 40 μg of protein (Western blotting) or pg of RXRα or RXRβ per μg of total protein (ELISAs). One-way analysis of variance (ANOVA) was preceded by the Levene test of homogeneity of variances and used to determine the overall significance. Differences between control and experimental groups were assessed using a post hoc Newman–Keuls test, and significant differences were designated as *p < 0.05, **p < 0.01, ***p < 0.001 (versus control cultures) and ^#^*p* < 0.05, ^##^*p* < 0.01, and ^###^*p* < 0.001 (versus the cultures exposed to DDE). The results were expressed as the mean ± SEM of three to four independent experiments. The number of replicates in each experiment ranged from 2 to 3, except for measurements of caspase-3 activity and LDH release, with replicates ranging from 5 to 8. To compare the effects of *p,p*′-DDE and *o,p*′-DDE in various brain tissues and different treatment paradigms, the results corresponding to caspase-3, LDH, DNA methylation and Western blot analysis were presented as a percentage of the control.

## Results

### Effects of DDE on Caspase-3 Activity and LDH Release in Hippocampal Cultures

#### Effects of *p,p*′-DDE on Caspase-3 Activity and LDH Release

**Fig. 1:**
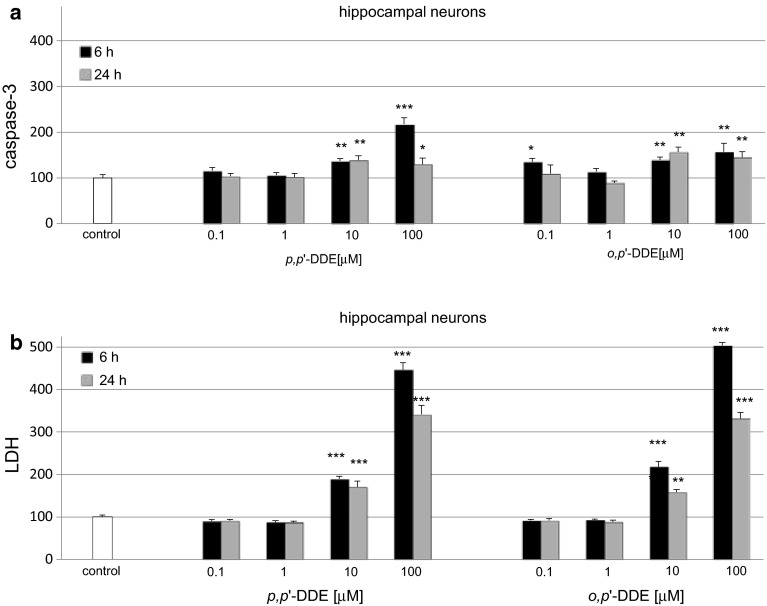
Time-course effects of *p,p*′-DDE and *o,p*′-DDE (0.1, 1, 10, and 100 μM) on caspase-3 activity (**a**) and LDH release (**b**) in primary cultures of mouse hippocampal cells at 7 DIV. Cells were treated with *p,p*′-DDE or *o,p*′-DDE for 6 and 24 h. The results are presented as a percentage of the control. Each *bar* represents the mean of three to four independent experiments ± SEM. The number of replicates in each experiment ranged from 5 to 8. *p < 0.05, **p < 0.01 and ***p < 0.001 versus control cultures

In hippocampal cultures exposed to 0.1–100 μM *p,p*′-DDE, the activity of caspase-3 increased to 220 and 140 % at 6 and 24 h, respectively (Fig. 1a). LDH release increased with the duration of *p,p*′-DDE treatment and was elevated to 450 and 340 % of the vehicle control at 6 and 24 h, respectively (Fig. 1b).

#### Effects of *o*,*p*′-DDE on Caspase-3 Activity and LDH Release

In hippocampal cultures exposed to 0.1–100 μM *o*,*p*′-DDE, the activity of caspase-3 increased to 160 % of the control level (Fig. 1a). LDH release significantly increased following exposure to *o*,*p*′-DDE for 6 h to 505 % and was elevated to over 300 % of the control at 24 h (Fig. 1b).

### Effects of DDE on Caspase-3 Activity and LDH Release in Neocortical Cultures

#### Effects of *p*,*p*′-DDE on Caspase-3 Activity and LDH Release

**Fig. 2:**
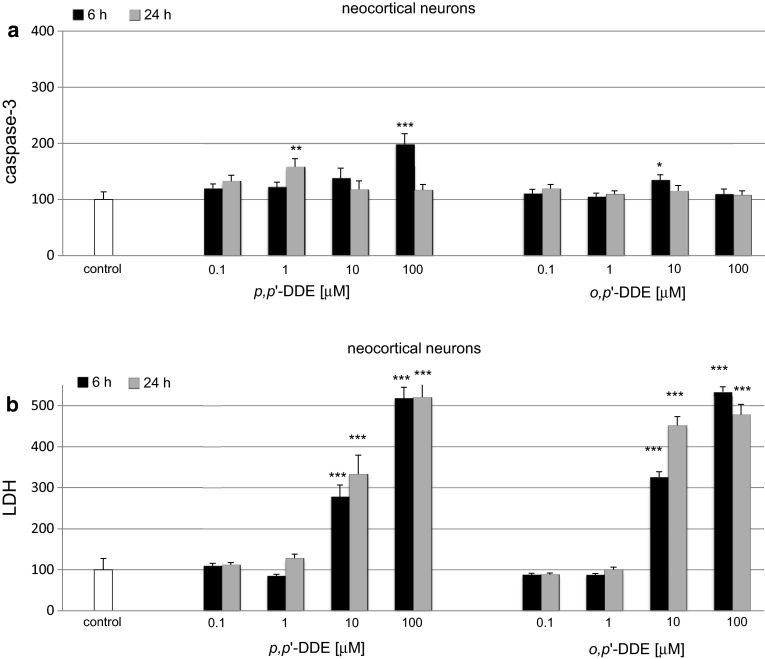
Time-course effects of *p,p*′-DDE and *o,p*′-DDE (0.1, 1, 10, 100 μM) on caspase-3 activity (**a**) and LDH release (**b**) in primary cultures of mouse neocortical cells at 7 DIV. The cells were treated with *p,p*′-DDE or *o,p*′-DDE for 6 and 24 h. The results are presented as a percentage of the control. Each *bar* represents the mean of three to four independent experiments ± SEM. The number of replicates in each experiment ranged from 5 to 8. *p < 0.05, **p < 0.01 and ***p < 0.001 versus control cultures

In neocortical cultures, *p,p*′-DDE (0.1–100 μM)-induced caspase-3 increased to 200 % of the control level at 6 h and remained enhanced to 160 % at 24 h post-treatment (Fig. 2a). In these cells, *p,p*′-DDE-induced caspase-3 activity was reduced to lower levels than those in hippocampal cells. LDH release values increased in neocortical cells to 280–520 % of the control value at 6 h and to 330–520 % at 24 h (Fig. 2b), and these values were similar to the LDH values indicated in hippocampal cultures.

#### Effects of *o*,*p*′-DDE on Caspase-3 Activity and LDH Release

In neocortical cultures, 0.1–100 μM *o,p*′-DDE the activity of caspase-3 increased to 135 % of the control level (Fig. 2a). The LDH release was elevated to 330–530 % of the control value at 6 h and to 450–480 % at 24 h (Fig. 2b). The effects observed in neocortical cell cultures were lower than those in the hippocampal cells but only in respect to caspase-3 activity. LDH values were similar in both types of cell cultures.

### Effects of DDE on Caspase-3 Activity and LDH Release in Cerebellar Cultures

#### Effects of *p*,*p*′-DDE on Caspase-3 Activity and LDH Release

**Fig. 3:**
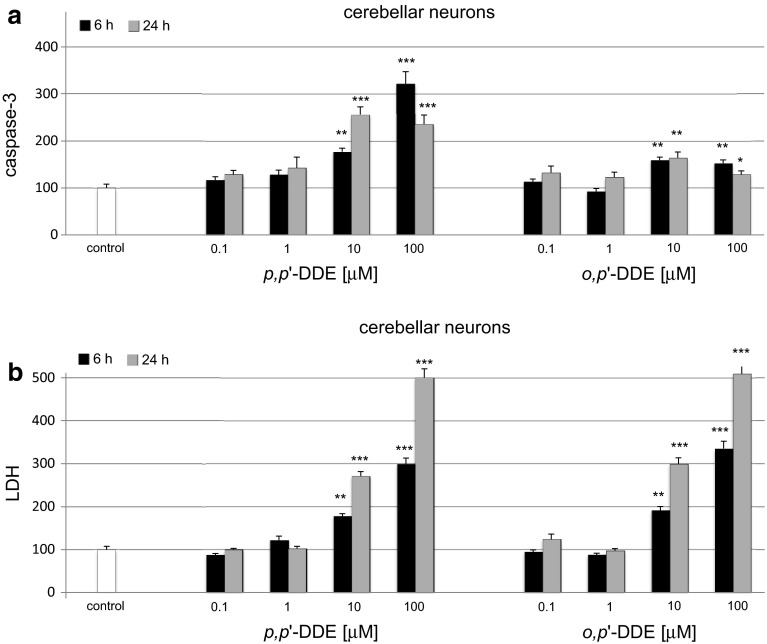
Time-course effects of *p,p*′-DDE and *o,p*′-DDE (0.1, 1, 10, 100 μM) on caspase-3 activity (**a**) and LDH release (**b**) in primary cultures of mouse cerebellar cells at 7 DIV. The cells were treated with *p,p*′-DDE or *o,p*′-DDE for 6 and 24 h. The results are presented as a percentage of the control. Each *bar* represents the mean of three to four independent experiments ± SEM. The number of replicates in each experiment ranged from 5 to 8. *p < 0.05, **p < 0.01 and ***p < 0.001 versus control cultures

In cerebellar cultures, the level of *p,p*′-DDE (0.1–100 μM)-activated caspase-3 ranged from 180 to 320 % and 235–260 % of the control value at 6 and 24 h, respectively (Fig. 3a). The *p,p*′-DDE-induced LDH release was enhanced to 180–300 % of the control level at 6 h and subsequently increased to 270–500 % at 24 h (Fig. 3b). The effects of 10 µM DDE observed in cerebellar cultures at 6 h of exposure were similar to those observed in hippocampal cultures, particularly in respect to *o,p*′-DDE. The LDH values were similar in both types of cell cultures.

#### Effects of *o*,*p*′-DDE on Caspase-3 Activity and LDH Release

In cerebellar cultures treated with 0.1–100 μM *o,p*′-DDE, the activity of caspase-3 increased to approximately by 160 % of the control value at 6 and 24 h (Fig. 3a). The LDH release was elevated to 335 % at 6 h and over 500 % at 24 h of the control value (Fig. 3b). The effects observed in cerebellar cell cultures were similar to those in the hippocampal cells, with respect to caspase-3 activity and LDH release.

### Effects of *p,p*′-DDE Alone or in Combination with HX 531 on Hoechst 33342 and Calcein AM Staining Experiments in Hippocampal Cultures

**Fig. 4:**
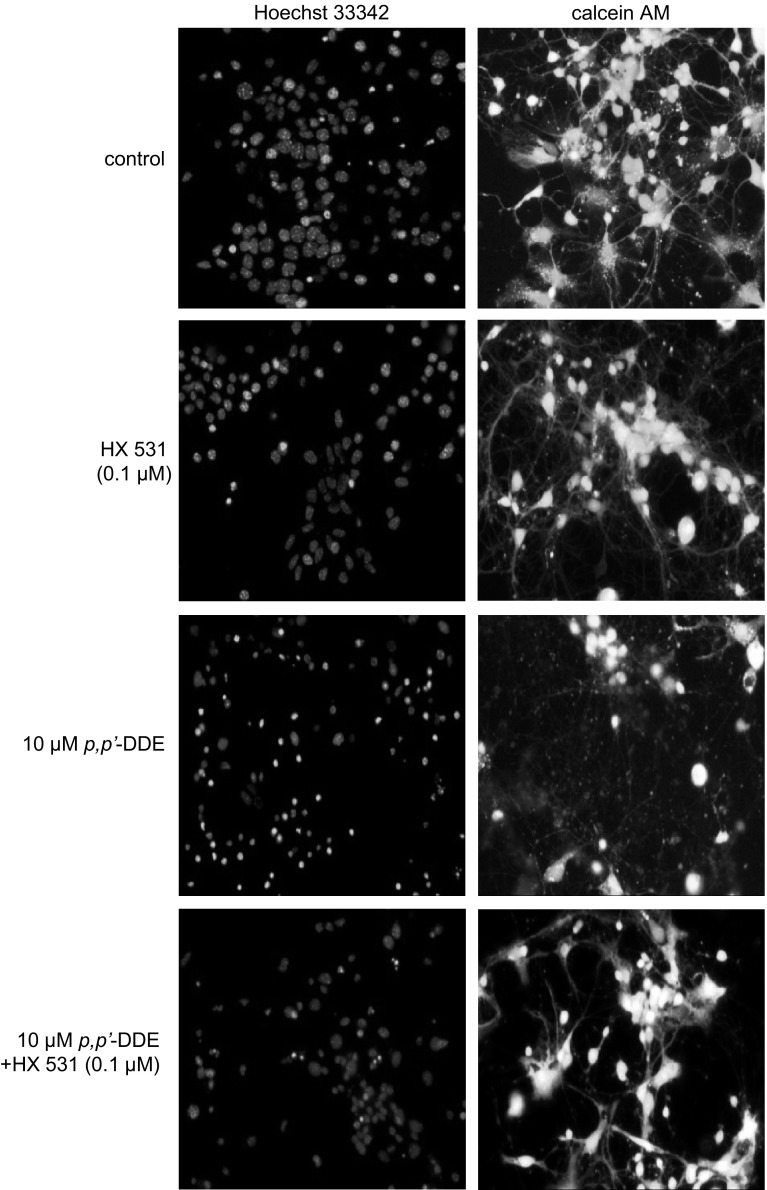
Influence of *p,p*′-DDE (10 μM) and HX 531 (0.1 μM) on Hoechst 33342 (*1st column*) and calcein AM (*2nd column*) staining in mouse hippocampal cultures at 7 DIV, examined 24 h post-treatment. The RXR antagonist HX 531 was added into the culture media approximately 45–60 min before *p,p*′-DDE was added. The cells were cultured on glass cover slips, washed with 10 mM PBS, and exposed to Hoechst 33342 (0.6 μg/ml) at RT for 5 min. The cells were subsequently rewashed and incubated with 2 μM calcein AM at RT for 10 min. Cells with bright fragmented nuclei showing condensed chromatin were identified as undergoing apoptosis, whereas cells with *light-colored* cytoplasm were identified as living cells (Color figure online)

A continuous 24-h exposure of hippocampal cultures to *p,p*′-DDE (10 μM) induced apoptosis in mouse hippocampal cells, evidenced by Hoechst 33342 staining. The formation of apoptotic bodies appeared as bright blue fragmented nuclei containing condensed chromatin labeled with Hoechst 33342 (Fig. 4). Treatment with DDE also decreased the number of cells with light-colored cytoplasm, indicating a reduced density of living cells, evidenced by calcein AM. Co-treatment with HX 531 (0.1 μM) inhibited the *p,p*′-DDE-induced effects with respect to both parameters.

### Influence of *p,p*′-DDE and *o,p*′-DDE on Changes in Global DNA Methylation in Hippocampal Cultures

**Fig. 5:**
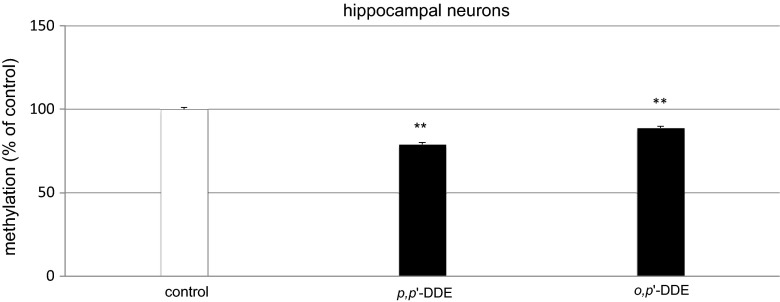
Influence of *p,p*′-DDE and *o,p*′-DDE on global DNA methylation at 7 DIV hippocampal cultures. Primary hippocampal cultures were treated with *p,p*′-DDE or *o,p*′-DDE (both at 10 μM) and total DNA was extracted from hippocampal cells at 24 h post-treatment, followed by ELISA. Each *bar* represents the mean of three independent experiments ± SEM. The number of replicates in each experiment ranged from 2 to 3. **p < 0.01 versus control cultures

The continuous 24-h exposure of hippocampal cultures to *p,p*′-DDE and *o,p*′-DDE caused changes in the level of global DNA methylation. The treatment with DDE decreased methylation level by 11–21 % of the control value, depending on DDE isomer (Fig. 5).

### Effect of DDE on mRNA Levels of *Rxrα*, *Rxrβ*, and *Rxrγ*

**Fig. 6:**
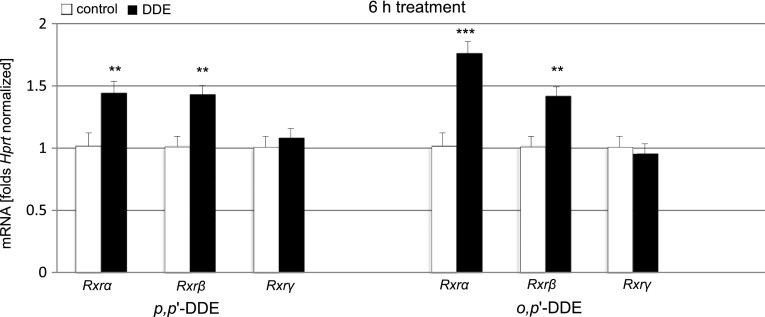
Effect of *p,p*′-DDE and *o,p*′-DDE (both at 10 μM) on the mRNA expression levels of *Rxrα*, *Rxrβ*, and *Rxrγ* in hippocampal cultures at 7 DIV. The extraction of total RNA at 6 h post-treatment from the hippocampal cells was followed by reverse transcription (RT) and quantitative polymerase chain reaction (qPCR). The products of the RT reaction were amplified using TaqMan probes and primers corresponding to the specific genes. *Hprt* was used as a reference gene. Each bar represents the mean ± SEM of three independent experiments. The number of replicates for each experiment ranged from 2 to 3, **p < 0.01, and ***p < 0.001 versus control cultures

Treatment with *p,p*′-DDE and *o,p*′-DDE (both 10 μM) evoked changes in mRNA levels of *Rxrα*, *Rxrβ*, and *Rxrγ*. A 6-h exposure of hippocampal cultures to *p,p*′-DDE caused a 42 % increase in *Rxrα* and a 41 % increase in *Rxrβ* but did not significantly affect *Rxrγ* mRNA compared with the control. Similarly *o,p*′-DDE induced a 73 % increase in *Rxrα* and a 40 % increase in *Rxrβ* mRNA compared with the control. In *o,p*′-DDE-treated cells, the *Rxrγ* mRNA level remained unchanged. These data were normalized to control *Hprt* (Fig. 6).

### Effects of DDE on the Protein Expression Levels of RXRα and RXRβ in Mouse Hippocampal Cells

**Fig. 7:**
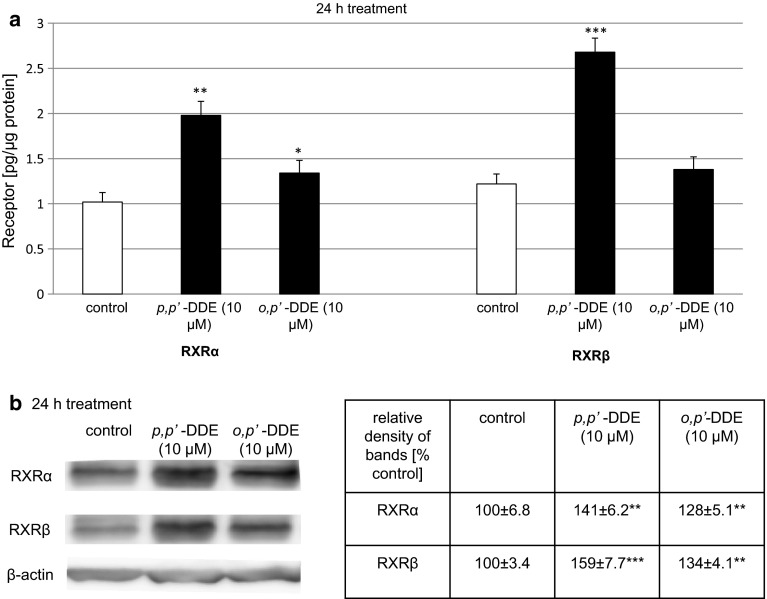
Effects of *p,p*′-DDE and *o,p*′-DDE on protein levels of RXRα and RXRβ in mouse hippocampal cultures at 7 DIV. Hippocampal cells were cultured for 7 DIV and then treated for 24 h with *p,p*′-DDE (10 μM) or *o,p*′-DDE (10 μM). The concentrations of the receptors were measured using specific ELISAs and presented as pg of RXRα or RXRβ per μg of total protein. For Western blot analyses, protein samples were denatured, electrophoretically separated, transferred to PVDF membrane, and subjected to immunolabeling. Signals were developed by chemiluminescence (ECL) and visualized with Luminescent Image Analyzer Fuji-Las 4000 (Fuji, Japan). Immunoreactive bands were quantified using an image analyzer (ScienceLab, MultiGauge V3.0), and the relative protein levels of RXRα and RXRβ were presented as a percentage of the control. Each *bar* or value represents the mean of three independent experiments ± SEM. The number of replicates in each experiment ranged from 2 to 3. *p < 0.05, **p < 0.01, and ***p < 0.001 versus control cultures

In this study, *Rxrγ* mRNA expression was not affected by *p,p*′- or *o,p*′-DDE. Therefore, in the following experiments, we measured only DDE-induced alterations in protein levels of RXRα and RXRβ. A 24-h exposure to DDE was necessary to detect changes in protein levels of the receptors. In the control cultures, RXRα and RXRβ reached 1.02 and 1.22 pg per μg of total protein, respectively. A 24-h exposure to *p,p*′-DDE (10 μM) nearly doubled the level of RXRα (1.98 pg/µg) and induced an increase in RXRβ of more than two times (2.68 pg/µg) (Fig. 7a). Hippocampal cells treated with *o,p*′-DDE (10 μM) increased RXRα and RXRβ levels by 13–31 % of the control values, i.e., approximately 1.35 pg/µg (Fig. 7a).

Western blot analysis demonstrated the constitutive protein expression of RXRα and RXRβ in mouse hippocampal cells at 7 DIV (Fig. 7b). Exposure to *p,p*′-DDE (10 μM) increased the relative RXRα and RXRβ protein levels by 41 and 59 %, respectively, at 24 h post-treatment. Treatment with *o,p*′-DDE (10 μM) increased the relative RXRα and RXRβ protein levels by 28 and 34 %, respectively (Fig. 7b).

### Impact of the RXR Antagonist on DDE-Induced Caspase-3 Activity and LDH Release in Hippocampal Cultures

#### Impact of the RXR Antagonist on *p,p*′-DDE-Induced Caspase-3 Activity and LDH Release

**Fig. 8:**
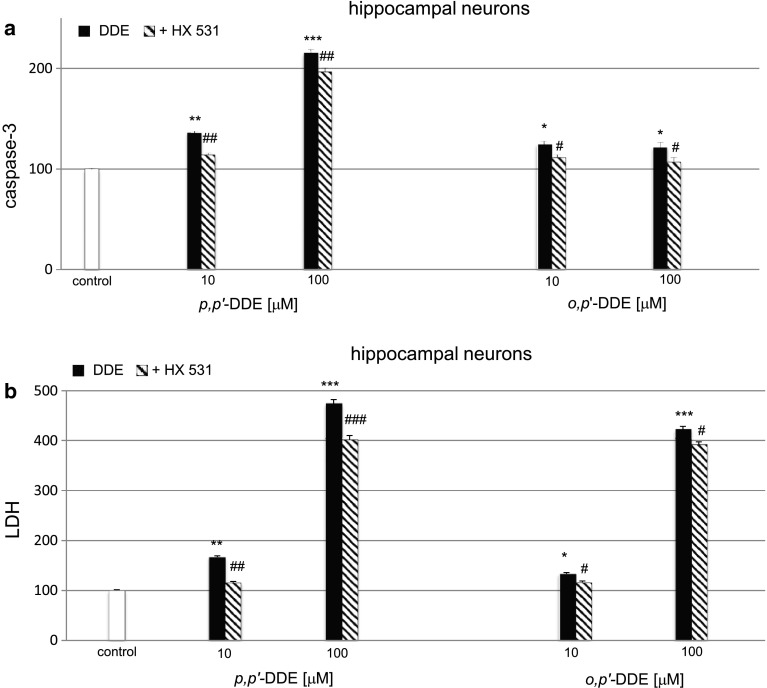
Impact of the RXR antagonist on *p,p*′-DDE and *o,p*′-DDE–induced on caspase-3 activity (**a**) and LDH release (**b**) at 7 DIV hippocampal cultures. Primary hippocampal cultures were treated with *p,p*′-DDE or *o,p*′-DDE (10, 100 μM) for 6 h. The RXR antagonist HX 531 (0.1 μM) was added into the culture media approximately 45–60 min before DDE was added. The results were normalized to the absorbency in vehicle-treated cells and expressed as a percentage of control. Each *bar* represents the mean of three to four independent experiments ± SEM. The number of replicates in each experiment ranged from 5 to 8. *p < 0.05, **p < 0.01, ***p < 0.001 versus control cultures, ^#^ *p* < 0.05, ^##^ *p* < 0.01, and ^###^ *p* < 0.001 versus the cultures exposed to DDE

We selected the 6 h treatment with DDE to study the effects of RXR antagonist. The RXR antagonist HX 531 (0.1 μM) inhibited *p,p*′-DDE (10 μM and 100 μM)-induced caspase-3 activity approximately by 20 % at 6 h (Fig. 8a) compared with control.

As demonstrated in panel b, 0.1 μM of the RXR antagonist HX 531 reduced LDH activity by 51–73 % in the cultures subjected to *p,p*′-DDE (10,100 μM) for 6 h (Fig. 8b).

#### Impact of the RXR Antagonist on *o,p*′-DDE-Induced Caspase-3 Activity and LDH Release

The *o,p*′-DDE (10 and 100 μM)-induced activation of caspase-3 was also inhibited approximately by 15 % at 6 h post-treatment using the RXR antagonist HX 531 (0.1 μM) (Fig. 8a). The effect of *o,p*′-DDE was also reduced by 17–31 % with respect to LDH activity in the presence of the HX 531 (Fig. 8b).

### Effect of DDE on the Distribution of RXRα, RXRβ, and MAP2 Staining in Hippocampal Cells

**Fig. 9:**
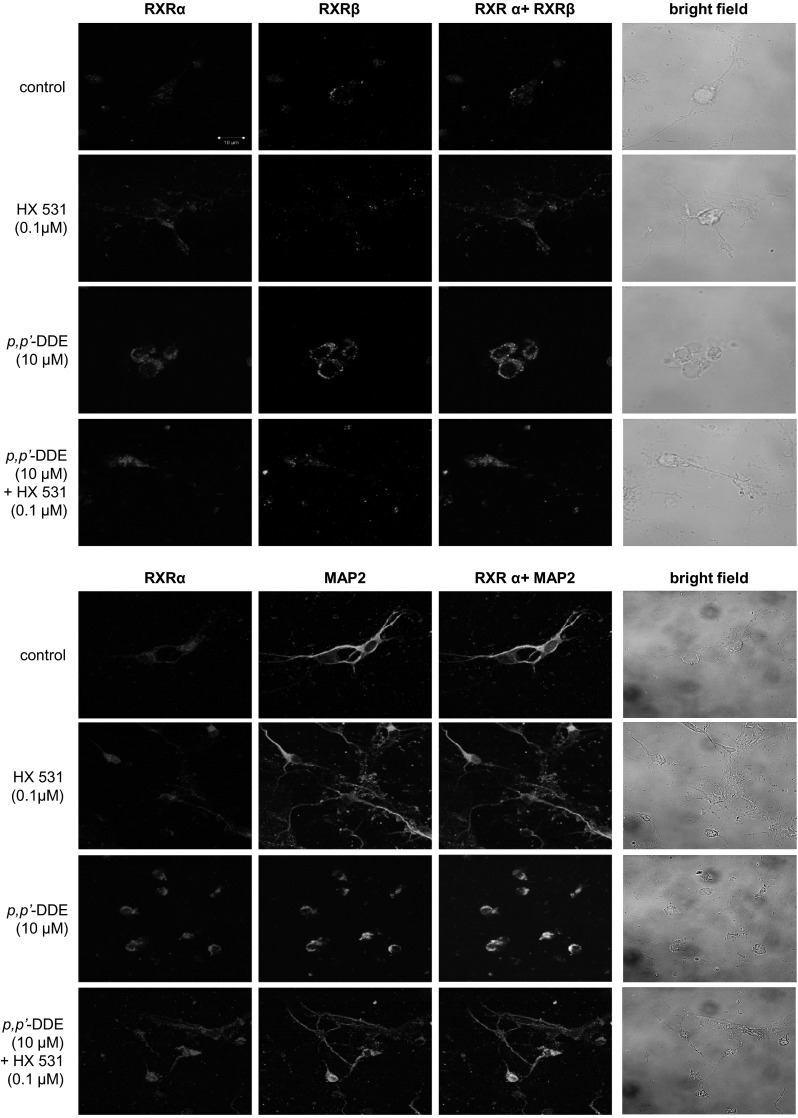
Influence of *p,p*′-DDE (10 μM) and HX 531 (0.1 μM) on the cellular distribution of RXRα (*red*), RXRβ (*blue*), and MAP2 (*blue*) in mouse hippocampal cultures at 7 DIV. The overlay of RXRα/RXRβ and RXRα/MAP2 (*blue* plus *red*) and *bright field* images are also shown. Primary hippocampal cultures were treated with *p,p*′-DDE (10 μM) alone or in combination with HX 531 for 24 h. The cells were cultured on glass cover slips and subjected to immunofluorescent double-labeling. The samples were analyzed using a confocal laser scanning spectral microscope LSM510 META, Axiovert 200 M (Carl Zeiss MicroImaging GmbH, Jena, Germany) with a Plan-Apochromat 63×/1.4 Oil DIC objective (Color figure online)

Immunofluorescence labeling and confocal microscopy revealed that RXRα and RXRβ were localized in the same hippocampal cells at 7 DIV (Fig. 9 upper panel). A 24-h exposure to *p,p*′-DDE (10 μM) increased the RXRα (red) and RXRβ (blue) staining. Treatment with HX 531 (0.1 μM) alone did not affect the levels of the receptors in the cell cultures. MAP2 staining confirmed the neural localization of RXR receptors and revealed the DDE-induced inhibition of neurite outgrowth (Fig. 9 lower panel).

### Influence of DDE on Caspase-3 Activity and LDH Release in Hippocampal Cells Transfected with RXRα and RXRβ siRNAs

**Fig. 10:**
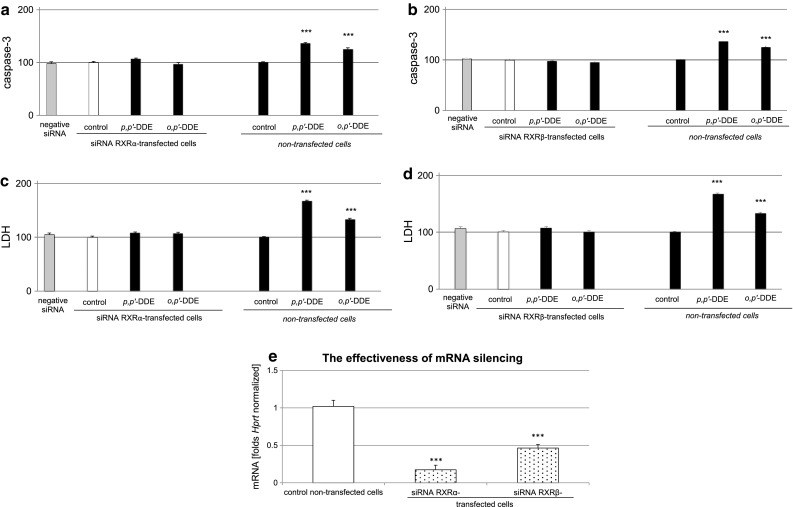
Effect of *p,p*′-DDE and *o,p*′-DDE (both at 10 μM) on caspase-3 activity (**a**, **b**) and LDH release (**c**, **d**) in RXRα or RXRβ siRNA-transfected hippocampal cells. The effectiveness of mRNA silencing was verified using qPCR (**e**). Primary hippocampal cultures were transfected with 50 nM RXRα or RXRβ siRNAs in INTERFERin™-containing medium without antibiotics for 6 h. The results were normalized to the absorbance in vehicle-treated cells and expressed as a percentage of the control, either in siRNA-transfected or non-transfected cells (**a**–**d**). mRNA silencing was presented as a fold of the control non-transfected cells (**e**). Each *bar* represents the mean ± SEM of three to four independent experiments. The number of replicates in each experiment ranged from 5 to 8. ***p < 0.001 versus the non-transfected control cultures

The pesticide was used at a 10 μM concentration to examine the actions of DDE in hippocampal cells transfected with RXRα and RXRβ siRNAs. A 6-h exposure to *p,p*′- and *o,p*′-DDE (10 µM) did not induce caspase-3 activity and LDH release in RXRα siRNA-transfected cells, suggesting that RXRα siRNA-transfected cells were less vulnerable to DDE than non-transfected cells (Fig. 10a, c). The effects of DDE in transfected cells were by 30 % lower than those in the non-transfected cells treated with DDEs with respect to caspase-3 and 27–59 % lower with respect to LDH.

RXRβ siRNA-transfected hippocampal cells did not respond to *p,p*′- or *o,p*′-DDE in terms of caspase-3 activity and LDH release. Thus, these cells were not vulnerable to DDE-induced apoptosis and neurotoxicity. Compared with non-transfected cells treated with DDEs, DDE-induced caspase-3 activity in transfected cells was reduced by 30–39 % and DDE-induced LDH release was reduced by 34–60 % (Fig. 10b, d).

The effectiveness of mRNA silencing was verified through the measurement of specific mRNAs using qPCR. In this study, mRNA silencing decreased the *Rxrα* concentration by 83 % (equal to 0.17-fold) and *Rxrβ* mRNA concentration by 62 % (equal to 0.38-fold); (Fig. 10e) compared to the non-transfected wild-type cells.

## Discussion

The results of the present study demonstrated the pro-apoptotic and toxic effects of DDT metabolites, i.e., *p,p*′-DDE and *o,p*′-DDE, in mouse embryonic neuronal cells. Both isomers evoked the concentration-dependent activation of caspase-3 and LDH release in the hippocampal, neocortical, and cerebellar tissues. In the paradigms examined in the present study, all types of brain tissue responded to only 10 and 100 μM *p,p*′- and *o,p*′-DDE. However, neocortical cells exhibited the weakest vulnerability to DDE isomers, particularly in terms of DDE-induced caspase-3 activity. Because DDE caused only weak activation of caspase-3 but induced substantial LDH release in the neocortical cell cultures, we suggest that apoptosis was only partly involved in DDE neurotoxicity in these cells. As for hippocampal and cerebellar cells, DDE led to high activation of caspase-3 which was reflected by increased levels of LDH, thus pointing to important role of apoptosis in the effects of DDE in the hippocampal and cerebellar cells. These biochemical alterations were accompanied by increased apoptotic body formation and impaired cell survival, evidenced by Hoechst 33342 and calcein AM staining. Bornman et al. (2007) previously observed the apoptotic effects of DDE in chicken embryonic neural cells (Bornman et al. 2007). Moreover, Shinomiya and Shinomiya (2003) demonstrated that *p,p*′-DDE (1–100 μM) induced apoptosis and suppressed neurite outgrowth in NGF-stimulated PC12 cells (Shinomiya et al. 2003).

In the present study, the highest concentration of DDE isomers caused extensive damage in neuronal tissues. Therefore, for subsequent experiments, we used a 10 μM concentration of the DDEs, which induced similar effects in hippocampal and cerebellar neurons after 6-h exposure. Similarly, 10 µM or even higher concentrations have been used for studying toxic effects of DDE in Sertoli cells, CAMA-1, and MCF-7 breast cancer cells as well as in human prostate cancer cells (Liang et al. 2008; Aubé et al. 2008; Wong et al. 2015). Furthermore, DDE has been found in human adipose tissue in concentration ranging from 1.49 to 2.33 mg/kg, which is equal to approximately 5–7 µM (Waliszewski et al. 2014). Based on epidemiological studies, increased levels of DDE have been reported in post-mortem Parkinson’s and Alzheimer’s diseases brains in U.S. and U.K. populations (Fleming et al. 1994; Corrigan et al. 1998, Richardson et al. 2014). According to Corrigan et al. 1998, mean concentration of DDE in Parkinson’s disease brain was 0.995 μg/g lipid (the highest level reached value of 15.81 μg/g lipid). In a previous study, we observed that hippocampal tissue was highly sensitive to the parental compound DDT (Kajta et al. 2014); therefore, we used hippocampal neuronal cells to examine the mechanisms underlying *p,p*′-DDE and *o,p*′-DDE activity. The results suggested that DDE evoked concentration- and tissue-dependent but not isomer-specific effects in mouse embryonic neuronal cells. The involvement of apoptosis in actions of DDE has been supported by measurements of caspase-3 and detection of apoptotic fragmentation of cell nuclei. In addition, the neuronal cell damage has been assessed in terms of lactate dehydrogenase release as well as calcein AM and MAP2 stainings.

In addition to the demonstration that DDE-induced apoptosis as evidenced by caspase-3 activation and apoptotic fragmentation of cell nuclei, we showed that *p,p*′- and *o,p*′-DDE inhibited global DNA methylation in mouse embryonic neuronal cells. Aberrant DNA methylation has been implicated as an epigenetic mechanism associated with a wide range of diseases, including those associated with exposure to environmental pollutants. Recently, it has been suggested that low doses of DDT might induce the incomplete methylation of specific gene regions in the young brain and impair hippocampal neurogenesis (Shutoh et al. 2009). The global DNA hypomethylation observed in the present study typically reflects chromosomal instability, reactivation of retrotransposable elements, and the expression of genes that would normally be silenced through methylation. One may assume that the DDE-induced global DNA hypomethylation observed in our study may have interfered with transcriptional status of specific apoptosis-related genes.

In the present study, we demonstrated that 10 µM *p,p*′- and *o,p*′-DDE stimulated the mRNA expression levels of *Rxrα* and *Rxrβ* but not *Rxrγ*. In addition, we provided evidence that DDE isomers enhanced protein levels of RXR receptors, detected using specific ELISA and Western blot analyses. We demonstrated that DDE-induced patterns of *Rxrα* and *Rxrβ* mRNA expression reflected alterations in the protein levels of the RXR receptors. Although our studies demonstrated pro-apoptotic and neurotoxic properties of RXRs, animal models of neural degenerations indicated that RXR activation improves neuronal survival (Riancho et al. 2015). These opposite effects are probably due to variety of heterodimerization partners of RXRs which may either mediate pro-survival or apoptotic pathways. RXRs are xenobiotic receptors involved in the propagation of xenobiotic-induced effects, including NP, TBT, TPT, bisphenol A (BPA), methoprene acid, and arsenite (Kanayama et al. 2005; Litwa et al. 2014; Nishizawa et al. 2005; Li et al. 2008). Recently, Li et al. 2008 showed that *p,p*′-DDE induced *RXR*-gene activity using a two-hydrid assay (Li et al. 2008). This assay employed recombined human *RXR* and reporter genes, which specifically expressed β-galactosidase when incubated with exogenous 9-*cis* retinoic acid. Although these authors observed the agonistic potency of *p,p*′-DDE toward RXR at a 1 µM concentration, these experiments were performed with yeasts and not neuronal cells. In the present study, both DDE isomers in 10 µM concentration substantially increased the mRNA expression levels of *Rxr* receptors, except *Rxrγ* mRNA. We also recently showed that NP did not affect *Rxrγ* mRNA in mouse hippocampal neurons (Litwa et al. 2014). Similarly, Shiizaki et al. (2014) provided a list of compounds that did not alter *Rxrγ* gene expression (Shiizaki et al. 2014). However, there are no relevant data from other researchers to compare with the DDE-stimulated protein levels of RXR receptors observed in the present study.

In addition, in this study, we showed that the potent RXRα/β antagonist HX 531 inhibited DDE-induced caspase-3 activity and LDH release, thus supporting RXR-mediated effects of DDE in mouse neuronal hippocampal cells. Because HX 531 antagonized the effects of DDE isomers only partially, we verified the involvement of particular RXRs in DDE-induced effects using specific siRNAs. RXRs are known to form heterodimers with various receptors including PXR, CAR, PPAR, Nurr1, Nurr77, LXR, VDR, and RAR. Although the above receptors can mediate different effects, they may also share competences. Up to now, no signaling pathway that is specific strictly for RXR has been identified. Therefore, in addition to RXR antagonist HX 531, we used specific RXR siRNAs. In contrast to non-transfected neuronal cells, siRNA-transfected cells did not respond to *p,p*′- and *o,p*′- DDEs, providing evidence on a key role for RXRα and RXRβ-signaling in the apoptotic and neurotoxic actions of both DDE isomers in mouse hippocampal cells. We also studied morphological alterations, evidenced as immunofluorescent labeling of RXRα, RXRβ and MAP2, only in response to *p,p*′-DDE. According to these data, *p,p*′-DDE treatment stimulated RXRα and RXRβ staining and reduced neurites. Although HX 531 did not entirely reverse the effect of the pesticide metabolite, this antagonist inhibited the *p,p*′-DDE-induced impairment of neuronal morphology. In our study, DDE did not affect the cellular distribution of the receptors in mouse hippocampal cells. One may assume that a 24-h of exposure was sufficient to detect increased protein levels of RXRα and RXRβ as evidenced by ELISAs and WB, but it was too long to notice the translocation of activated receptors from cytoplasm to the nuclei.

In summary, the results of the present study demonstrated that *p,p*′-DDE, and *o,p*′-DDE induced a caspase-3-dependent apoptosis and caused the global DNA hypomethylation in mouse embryonic neuronal cells. These results also provided evidence for DDE-isomer-non-specific alterations of RXRα- and RXRβ-mediated intracellular signaling, including changes in the receptor mRNA and protein levels. DDE-induced stimulation of RXRα and RXRβ was verified using a potent antagonist and specific siRNAs. The co-localization of RXRα and RXRβ was demonstrated using confocal microscopy. The apoptotic action of DDE was supported at the cellular level through Hoechst 33342 and calcein AM staining experiments.

## Conclusions

This study demonstrates that the stimulation of RXRα- and RXRβ-mediated intracellular signaling plays an important role in the propagation of DDE-induced apoptosis during the early stages of neural development.

## Abbreviations

ADHD: Attention deficit hyperactivity disorder
ALS: Amyotrophic lateral sclerosis
ANOVA: Analysis of variance
BPA: Bisphenol A
DA: Dopaminergic neurons
DDE: Dichlorodiphenyldichloroethylene
DDT: Dichlorodiphenyltrichloroethane
DHA: Docosahexaenoic acid
DIV: Days in vitro
ELISA: Enzyme-linked immunosorbent assay
Hprt: Hypoxanthine-guanine phosphoribosyltransferase
LDH: Lactate dehydrogenase
LTD: Long-term depression
LTP: Long-term potentiation
NP: Nonylphenol
PBS: Phosphate-buffered saline
PPAR: Peroxisome proliferator-activated receptors
qPCR: Quantitative polymerase chain reaction
RA: Retinoic acid
RAR: Retinoic acid receptor
RT: Room temperature/reverse transcription
RXR: Retinoid X receptor
T3R: Thyroid hormone receptor
TBT: Tributyltin
TPT: Triphenyltin
VDR: Vitamin D receptor
